# Behavior of YSZ (High Y_2_O_3_ Content) Layer on Inconel to Electro-Chemical Corrosion

**DOI:** 10.3390/ma18020400

**Published:** 2025-01-16

**Authors:** Ionut Adomniței, Ramona Cimpoeșu, Daniela Lucia Chicet, Margareta Coteață, Fabian Cezar Lupu, Costică Bejinariu, Liviu Andrușcă, Petronela Paraschiv, Mihai Axinte, Gheorghe Bădărău, Nicanor Cimpoeșu

**Affiliations:** 1 Faculty of Materials Science and Engineering, “Gheorghe Asachi” Technical University of Iasi, 67 Dimitrie Mangeron Street, 700050 Iasi, Romania; ionut.adomnitei@student.tuiasi.ro (I.A.); ramona.cimpoesu@academic.tuiasi.ro (R.C.); mihai.axinte@academic.tuiasi.ro (M.A.); gheorghe.badarau@academic.tuiasi.ro (G.B.); nicanor.cimpoesu@academic.tuiasi.ro (N.C.); 2 Faculty of Machine Manufacturing and Industrial Management, “Gheorghe Asachi” Technical University of Iasi, 700050 Iasi, Romania; margareta.coteata@academic.tuiasi.ro (M.C.); petronela.paraschiv@academic.tuiasi.ro (P.P.); 3 Faculty of Mechanical Engineering, “Gheorghe Asachi” Technical University of Iasi, 43 Dimitrie Mangeron Blvd., 700050 Iasi, Romania; fabian-cezar.lupu@academic.tuiasi.ro (F.C.L.); liviu.andrusca@academic.tuiasi.ro (L.A.); 4 Academy of Romanian Scientists, Ilfov 3, 050044 Bucharest, Romania

**Keywords:** Inconel, ceramic coating, electro-chemical corrosion, microstructure

## Abstract

The high yttria content of a stabilized zirconia (YSZ) (38 wt% Y_2_O_3_) coating was deposited by atmospheric plasma spraying (APS) from Metco 207 powders on an Inconel 718 (Ni-based superalloy) substrate. As a metal coating connection, a layer of cermet powder (Ni-20% Al—410NS) was used before the ceramic layer deposition. The electro-chemical corrosion resistance of these materials was tested using Inconel cylinders with a diameter of 10 mm and a thickness of 1 mm, with and without the ceramic layer. Linear and cyclic measurements were obtained in H_2_SO_4_ electrolyte media at pH = 2. Electro-impedance spectroscopy (EIS) experiments were performed on the sample covered with the ceramic layer to evaluate the interface behavior. Scanning electron microscopy (SEM), along with equipment to determine chemical composition, and an energy dispersive spectrometry (EDS) detector were used to characterize the material surface before and after corrosion tests. It was observed that the corrosion resistance of Inconel was influenced by the bonding layer and the ceramic coating.

## 1. Introduction

Ceramic layers deposited on Inconel 718 fulfill several important roles, especially in high-temperature and highly corrosive environments. In the case of thermal barriers, the ceramic layer acts as an insulator, protecting the base metal from extreme temperatures. This is crucial in applications such as gas turbines, where the components are exposed to high heat [1,2,3]. Inconel alloy 718 is already known for its excellent corrosion resistance, but the addition of a ceramic coating enhances this property. It provides a barrier against excessive oxidation and other corrosive agents [4,5]. The wear resistance of the ceramic layer can significantly improve the endurance of Inconel 718 components, making them suitable for applications involving abrasive environments [6]. By using a ceramic coating instead of heavier materials for protection, the overall weight can be reduced, which is particularly beneficial in aerospace applications. The service life is improved: the combination of thermal insulation and corrosion and wear resistance helps to extend the operational life of components, reducing maintenance and replacement costs [7,8,9].

Atmospheric plasma spraying (APS) is a widely used method for depositing ceramic coatings. The following are some advantages of using this technique for ceramic coatings: APS can reach very high temperatures, allowing the melting and deposition of a wide range of ceramic materials; the process can work with various types of ceramic materials, including oxides, nitrides, and carbides, making it suitable for various applications (versatility); the high kinetic energy of the particles during deposition leads to a strong mechanical adhesion between the coating and the substrate, enhancing durability (very good adhesion); APS enables the deposition of coatings with uniform and controllable thicknesses on complex geometries (uniformity); the process is relatively fast, which can lead to increased productivity and reduced production costs (productivity); and by adjusting parameters such as the base material, particle size, and spray distance, the properties of the coatings can be tailored to meet specific requirements [10,11].

Pavithran et al. conducted a study aimed at improving the corrosion resistance and thermal behavior of the Inconel 718 additively manufactured alloy using a yttria-stabilized zirconia (YSZ with 7 wt% Y_2_O_3_) thermal barrier layer. The results showed that the YSZ thermal barrier coating was essential for protecting Inconel 718 in aggressive corrosive and thermal environments, improving performance and lifetime for high temperature applications [12,13]. Most corrosion resistance tests of YSZ (7 wt% Y_2_O_3_) ceramic couplings were performed in CMAS (CaMgAlSi) media with different salt additions and at different temperatures [14,15,16,17]. As a new tool, Barbero and Hernández-Acosta proposed fractional calculus for electrical impedance evaluation in complex systems [18,19].

Composite ceramics with YSZ were obtained as thermal barriers and were reported to have very good results [20,21]. For composite ceramic coating growth through APS, all the investigated samples showed a similar phase composition, with tetragonal yttrium zirconium oxide (YZO—Y0.077Z0.923O1.962) and a mixture of crystalline and amorphous gamma alumina phase (γ-Al_2_O_3_) corundum (α-Al_2_O_3_) [22,23,24].

In this paper, the authors evaluated the corrosion resistance of new plasma spray deposited ceramic coatings. Their main characteristic is high stability at high temperatures as a result of the higher yttria content, which leads to zirconia stabilization.

## 2. Materials and Methods

Samples of commercially available Inconel 718 (Standard DIN 24668, WTE Power Steel, Český Brod, Czech Republic, Europe, tensile strength Rm: from 1241 MPa; yield strength: from 1034 MPa) were wire cut into samples with a diameter of 8 mm and a thickness of 3 mm for electro-corrosion resistance determination [25]. All the materials were mechanically polished in order to remove the oxides and impurities from the surfaces. Coated samples using atmospheric plasma spraying (APS) were obtained from Metco powders (Metco 410 for bond layer and of YSZ with 38% Y_2_O_3_ for zirconia stabilization—Metco 207). The bonding layer, which was first deposited on the Inconel substrate, was realized by two successive passes. The deposition was performed with a Sulzer Metco 9MCE, Sulzer Metco (Oerlikon), Wohlen, Switzerland equipped with a robotic arm. The operating parameters were 500 A and 62 V, and the powder temperature reached about 10,000 °C. Argon was used as primary gas and hydrogen and helium as secondary gases. The spray distance between the gun and the substrate was 120 mm [26].

**Figure 1:**
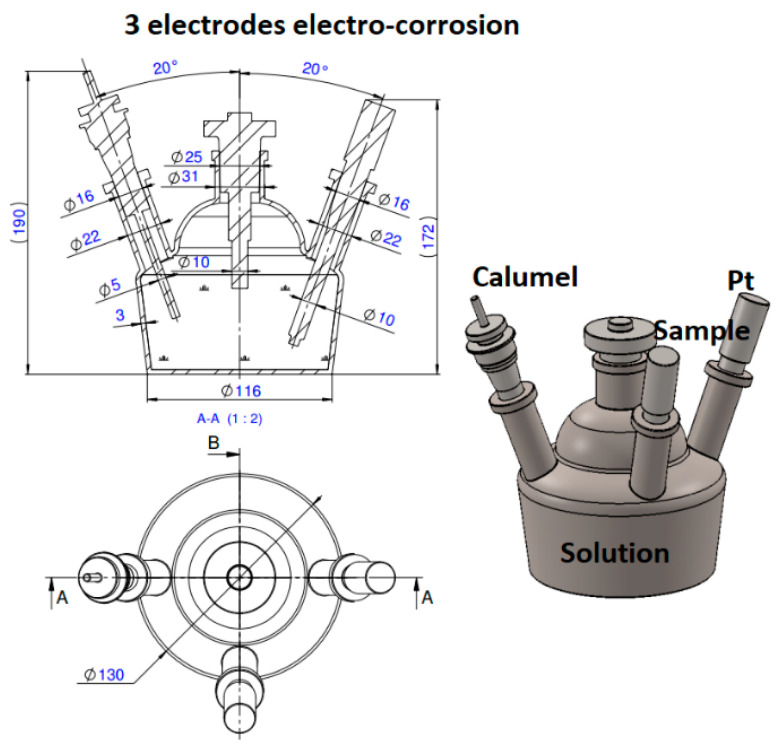
Electro-chemical corrosion cell.

For electrochemical measurements, an OrigaFlex—OGF01A potentiostat, OrigaLys, Rillieux-la-Pape, France equipped with a 3-electrode cell, a platinum auxiliary electrode, a reference calomel, and a working electrode with an exposed area of 0.5 cm^2^ was used, as shown in Figure 1. Linear and cyclic potentiometry were recorded in electrolyte media of H_2_SO_4_ with pH = 2. Linear potentiometry was recorded over a range of ±400 mV and linear potentiometry of −200–+600 mV. Data and Tafel plots were obtained using Origam Master5 software.

The working conditions used in the measurements were as follows:

- Linear anodic polarization for Tafel method: potential range (−400)–(+400) mV for the open-circuit potential, potential scanning rate: dE/dt = 0.1 mV/s;
- cyclic polarization: potential range (−200)–(+500) mV, potential scanning rate = 10 mV/s;
- EIS measurements: frequency range = 105–0.01 Hz, current amplitude = 10 mV [27,28].

The surfaces of the materials, including both the substrate and the substrate with ceramic coatings, were investigated before and after the electrochemical corrosion resistance test by scanning electron microscopy (SEM VegaTescan LMH II, SE detector, VegaTC, Brno, Czech Republic, 30 kV, 15 WD) and by X-ray energy spectroscopy (EDS detector from Bruker, Berlin, Germany, 6i/10, Esprit 2.2 software, automatic mode, mapping, and line features).

## 3. Results and Discussion

The experimental results are focused on the structural and chemical investigations of the ceramic layers deposited on the Inconel substrate, both on the surface and transversely, as well as on the electrochemical corrosion resistance of the Inconel superalloy with and without the ceramic coating.

### 3.1. Structural and Chemical Analysis of YSZ Coatings

**Figure 2:**
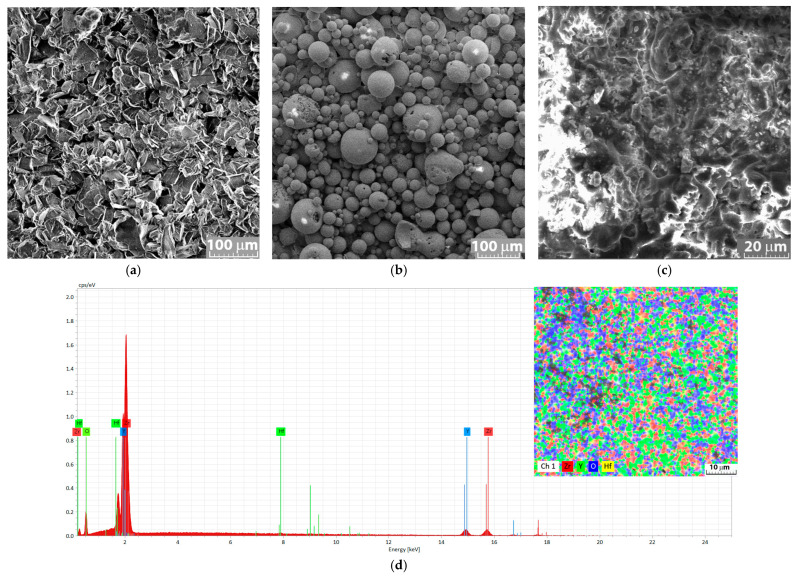
SEM images of (**a**) Metco 410 powders, (**b**) Metco 207 powders, (**c**) ceramic coating surface after APC, (**d**) chemical elements (energy spectrum) for qualitative identification and main element mapping on the coating.

For the coatings, NiAl (Ni 20% Al—chemical formula Al_2_O_3_ 30(Ni 20Al)) powders were used to grow the bond layer (Figure 2a) with dimensions of 75 ± 10 μm, with angular, blocky, or spheroidal shapes. YSZ powders with high yttria content (chemical formula: ZrO_2_-38Y_2_O_3_) (Figure 2b) were also used; these had a spherical morphology (d_min_ = 16 μm, d_max_ = 110 μm, d_mean_ = 35 μm ± 15). The ceramic coating exhibited a porous structure (Figure 2c), which is typical of APC ceramic coatings. The NiAl bond coat was chosen to improve the adhesion of the ceramic coating to the substrate (Ni-based material). Among the metal material options for existing bonding layers (such as Ni-Cr, NiCrAlY and CoCrAlY), Ni- and Al-based powders were chosen because of the Inconel substrate and the very good intermediate properties between materials with different expansion coefficients, such as metal and ceramic materials. The resulting coatings are also considered to be denser, harder, and more wear-resistant than conventional ceramic coatings. The surface of the ceramic coating (Figure 2c) showed a homogeneous ceramic layer consisting of molten particles with few pores and very few partially melted or partially solidified particles at the surface.

The energy spectrum (Esprit 2.2 software, automatic analysis mode, qualitative investigation) given in Figure 2d reveals the presence of Zr, Y, and O, which are specific to the YSZ compound, and Hf as a companion element identified on the surface (Figure 2c). No elements were identified in the bonding layer (neither Ni nor Al) or, normally, on the substrate, which confirms a very good coverage of the surface; this is to be expected in the case of thicker deposited layers, such as those acting as thermal barriers. It can also be stated that the pores in the deposited layers do not make a direct connection with the substrate or the bonding layer, which would lead to a significant reduction in the anti-corrosion protection of these ceramic coatings. From the surface distribution of the chemical elements (Figure 2d detail), a good chemical homogeneity of the formed ceramic layer was observed.

**Figure 3:**
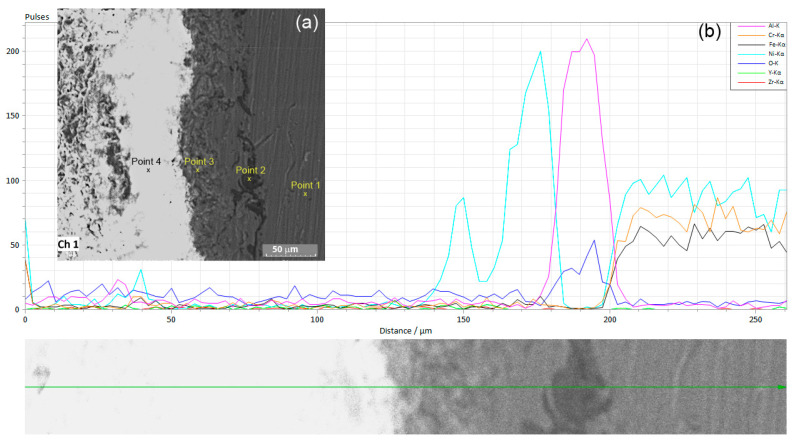
SEM image of the section in (**a**) and line distribution of the elements in (**b**).

**Figure 4:**
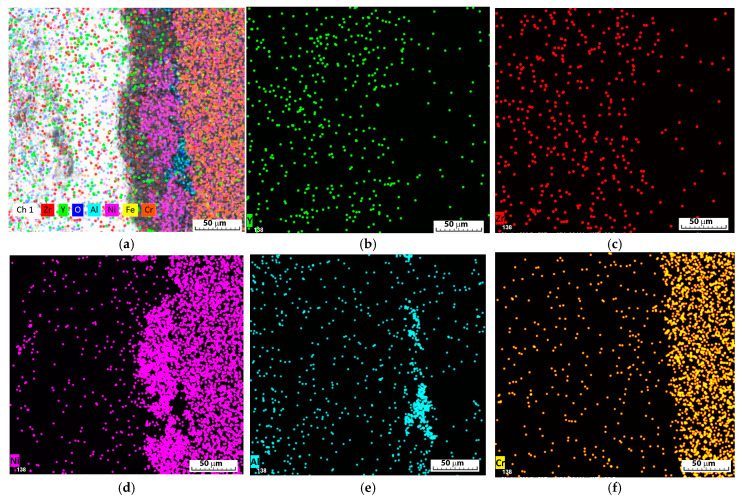
Main elements distribution: (**a**) all elements and (**b**) Y, (**c**) Zr, (**d**) Ni, (**e**) Al, and (**f**) Cr distributions on the surface from Figure 3a.

Structurally, four different zones can be seen in the cross-section (Figure 3a and Figure 4a): from right to left are the Inconel substrate, a thin darker layer, and a thicker layer that is still metallic in nature; fourth on the left is a layer of a non-metallic nature. Figure 3a shows a section of the coated Inconel; the left grey part is the ceramic coating, the middle is the bonding layer, and the right part is the substrate (the marked points are the areas used for chemical composition determinations). The variation in the chemical composition (Figure 3b and Figure 4) across the transversal sample confirmed the presence of the ceramic coating and of the bonding layer on the substrate. A very good adhesion of the deposited layers to the substrate was observed, without any interruption between them, cracks, or air gaps trapped at the interface.

The ceramic coating had a thickness of between 150 and 155 μm (Table 1, point 4); it was gathered after 10 passes during the deposition process (the passes were made successively from left to right and from top to bottom and vice-versa to sufficiently cover the entire surface of the substrate and the pores that form during deposition, in order to avoid the connection of the pores in the 10 overlapping layers and the formation of a path between the surface and the substrate as much as possible). The bonding layer had a thickness of around 60 μm, formed mostly from 45 μm of nickel (Table 1, point 3) and alumina of around 15 μm (Table 1, point 2, as a thin dark layer). As shown in Figure 3b, the line mode analysis of the main elements of the substrate, bonding layer, and ceramic coating confirmed the good adhesion between the Inconel substrate on the right part of the variation, the bonding layer, and the ceramic coating.

On top of the substrate, an irregular alumina layer appeared; it reached the substrate first, followed by a thicker Ni-based layer (Figure 3b and Figure 4b,d,e) and the ceramic coating where the substrate element signal was covered and Zr, O, and Y had a reduced signal. The nickel signal appears stronger in the bonding layer (Figure 3b) compared to the Ni bound in the Inconel, where the percentage of Ni is also smaller.

Due to the good coverage of the surface by the intercalation of the deposition direction of the layers, but also due to the greater number of ceramic layers deposited, the presence of the bonding layer does not affect the chemical composition of the ceramic layer surface (Table 1). Between the two samples, with and without the bonding layer, there are some very small differences in chemical composition, which fall within the limits of the EDS detector errors and within the standard deviations calculated for each chemical element; these were obtained from three determinations of the chemical composition of each element.

Moreover, since the chemical analyses were performed on arbitrary surfaces and on different areas and samples, it can be concluded that a very good chemical homogeneity of the ceramic layer was obtained.

### 3.2. Linear and Cyclic Potentiometry and Electro-Impedance Spectroscopy (EIS)

**Figure 5:**
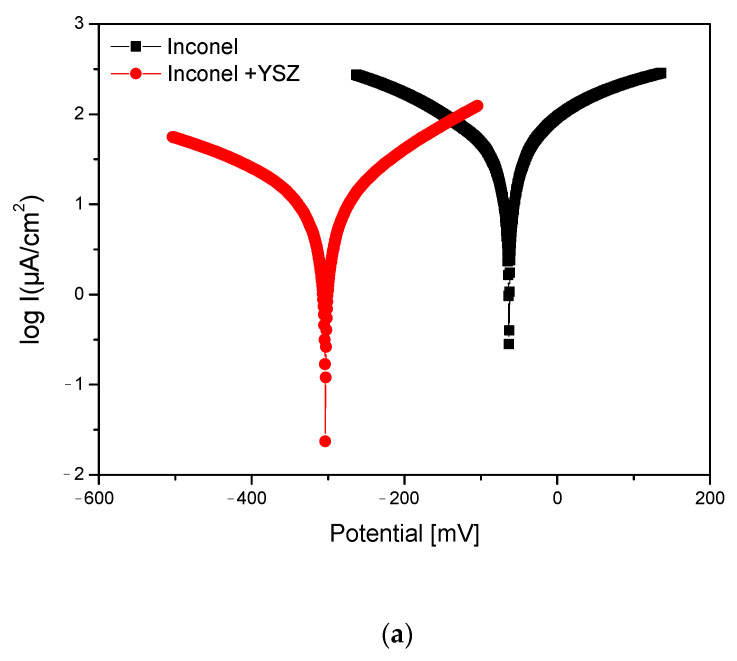

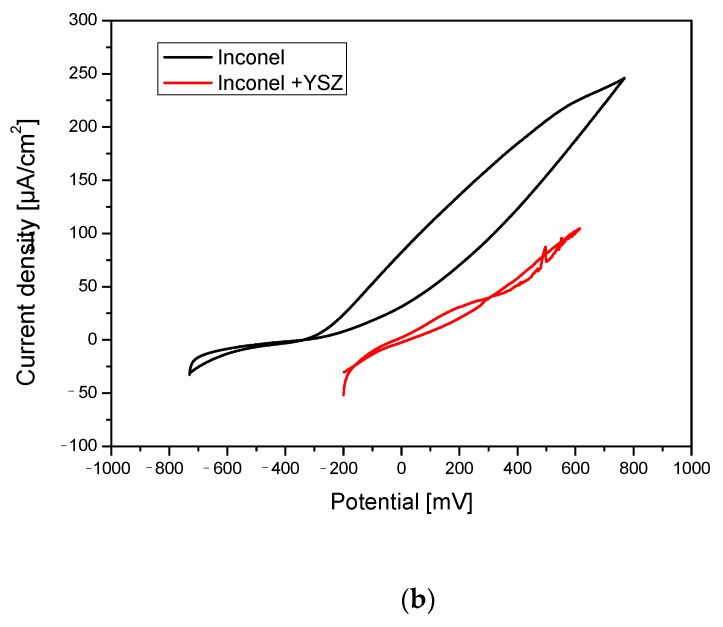
Tafel (**a**) and cyclic (**b**) diagrams of Inconel and Inconel + YSZ after the electro-corrosion test.

The experimental samples (Inconel with polished surface and Inconel + YSZ with polished surface) were tested for electrochemical corrosion resistance by linear and cyclic potentiometry tests in a sulfuric acid electrolyte solution with pH = 2; their variations are shown in Figure 5.

For the linear potentiometry, the electrochemical parameters were determined by Tafel extrapolation based on the ASTM G-102, 2015, USA standard. Figure 5a shows the variation in the corrosion current as a function of potential for the Inconel sample and for the Inconel coated with YSZ. Table 2 contains the main parameters extracted during the experiments (three determinations were made on samples with the same dimensions of exposed surfaces). Although the uncoated Inconel sample had a lower corrosion potential in the acidic solution compared to the coated one (Figure 5a), the corrosion current density was much lower for the sample coated with the ceramic layer (Table 2), resulting in a higher polarization resistance of this sample; it was about five times higher than that of Inconel; thus, it would contribute more to the total corrosion resistance in this electrolyte solution.

Since the corrosion rate can be obtained from the analysis of the corrosion current density using Faraday’s law, as given in Table 2, Equation (1) can be applied [29]:

v_corr_ = 0.00327 × Ew × i_corr_/D (1)

where D is the density of Inconel of 8.24 g/cm^3^, and Ew is the equivalent weight of the Inconel 718 alloy. A speed that was approximately seven times lower for the Inconel with ceramic coatings was calculated. Analyzing the values of the electrochemical corrosion parameters (Table 2), it was observed that the formation and growth of the protective passivation layer was achieved more easily for the sample with ceramic coatings. The ceramic material barrier deposited on Inconel had a protective role in preventing direct contact of the corrosive species with the substrate surface. This is also attributed to the very good adhesion of the YSZ layer to the Inconel substrate [12]; in particular, it is due to the deposited bonding layer, which contributes to the increase in the mechanical strength of Inconel and its resistance to higher temperatures (thermal shock resistance) [30].

The i_corr_ value (Table 2) for the uncoated substrate was several times higher. This was caused by the fact that the surface of the metal was exposed to the electrolyte solution and direct contact with oxygen without restriction, and anodic and cathodic electrochemical reactions (β_a_ and β_c_) occurred on its surface. Since some researchers consider the influence of the ceramic material, which is chemically inert, to be insignificant in the i_corr_ value, it can be concluded that the bond coat in this case also plays an important role in reducing the corrosion rate of the coated sample, especially the slight anodic reduction also observed from the β_a_ branch values [31]. The results obtained from the electrochemical corrosion (Table 2) were similar (i_corr_ and v_corr_) to those obtained for a coated Inconel 625 with a double layer of YSZ/NiCo grown by an electrophoretic process in a salt solution (3.5% NaCl) [32].

The interconnected and open surface porosity of plasma-sprayed coatings contributes to the contact of the electrolyte with the metal surface. Due to the ceramic nature of the coating, made in this case from two ceramic oxides with generally high corrosion resistance, ZrO_2_ and Y_2_O_3_, they exhibit excellent chemical stability and corrosion resistance in most corrosive media, such as H_2_SO_4_ solution [31,33].

Therefore, the chemical or electrochemical interactions between the sample and the electrolyte have little or no effect on the ceramic coating. Above a certain (higher) porosity, the coating becomes transparent to the electrolyte and only cathodically protects the substrate.

There was no active dissolution region in the acidic solution at pH = 2, which was followed by a passivation region; the alloys tested tended to passivate to an extent depending on the pH of the solutions. Within this region, as the potential increases slightly to positive, the current density decreases as a consequence of the deposition or formation of corrosion compounds on the surface and/or the growth of metal oxide films on the alloy surface. One of the behaviors of these films had a partially protective influence and reduced the active dissolution of the alloy (passivation). These results show that the oxide film was stable in this range of potentials, resulting in a very low current. Cyclic potentiometry presented the general corrosion character of the experimental samples in H_2_SO_4_. The surfaces showed that a passivation process was registered for these samples based on a continuous degradation. The current density presented a significant decrease for the sample coated with YSZ (Figure 5b). The presence of a few pores on the structure of the ceramic coating also modified the reverse curve of the cyclic potentiometry. The current density for the coated sample was reduced by half compared to the uncoated Inconel sample.

**Figure 6:**
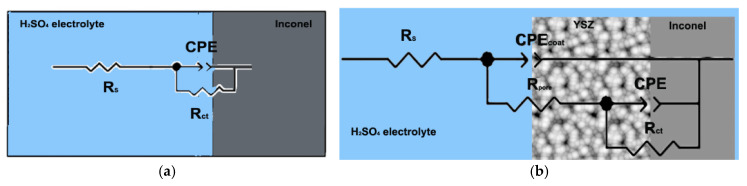
Fitting circuits of (**a**) Inconel and (**b**) Inconel + YSZ (chosen from the possible circuits of EIS software, ZSimpWin 3.22 database with the higher fitting score with the experimental results).

**Figure 7:**
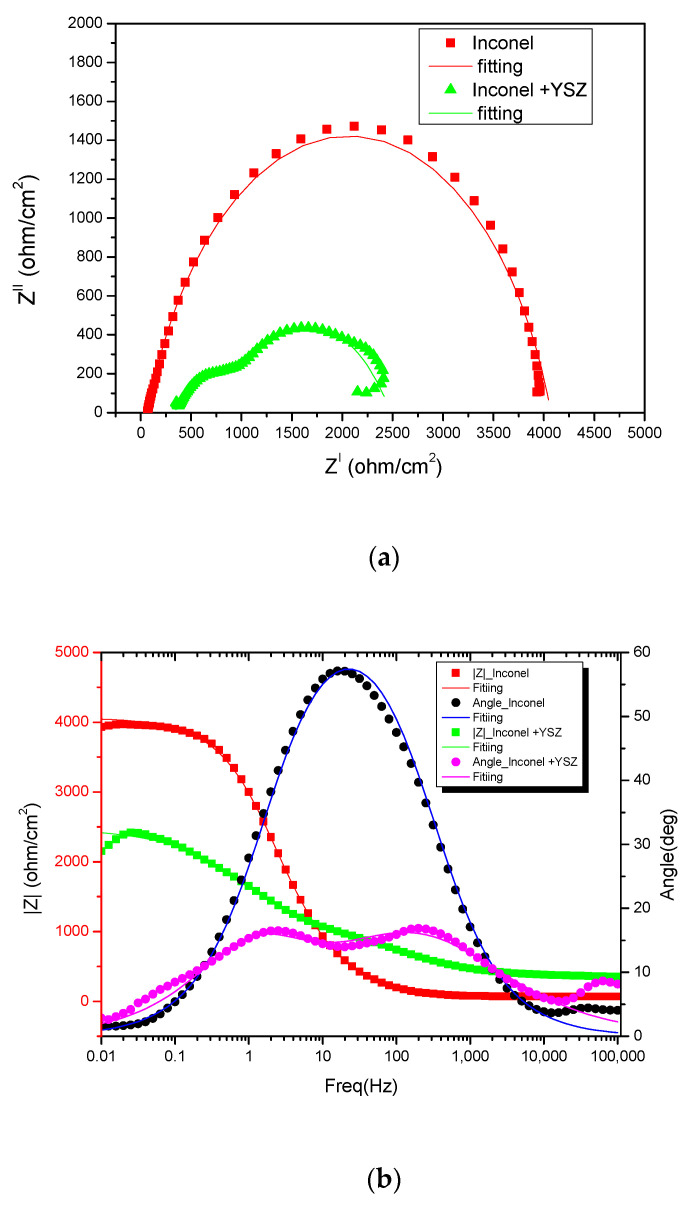
Nyquist plots in (**a**) and Bode plots in (**b**) of Inconel and Inconel + YSZ sample.

The Nyquist and Bode variations of the initial substrate (uncoated Inconel 718) are based on one time constant, whereas two time constants were used for the ceramic coated material. Two electrical circuits—EC (equivalent) (Figure 6) based on the above explanations and the number of time constants in the Nyquist and Bode plots in Figure 7, were proposed for the corrosion studies of the samples. In Figure 6a, the equivalent circuit of the experiment (electrochemical system) between the Inconel (substrate) and acidic solution (electrolyte) is shown: R_s_: a compensated resistance between working and reference electrodes; R_ct_: a resistance attributed to the charge transfer in the electrical double layer (EDL) at the interface of the substrate and solution; CPE_dl_ is a constant phase element of the double layer replacing the capacitance found in the electrical circuits.

In the second case, in addition to the substrate and the solution, the ceramic coating has to be considered (Figure 6b). In the second circuit, along with the aforementioned elements, R_pore_ was inserted: pore resistance in the coating, as was CPE_dl_: capacitance in the EDL at the interface of the substrate and the solution and CPE_coat_: capacitance of the coating. Figure 7a shows the Nyquist plots obtained from the EIS test for the Inconel and Inconel + YSZ coatings in acid electrolyte solution. The numerical values extracted from both samples are given in Table 3. As described in Figure 7a, the Inconel substrate in the EIS experiment consists of a single time constant corresponding to the electrical double layer on its surface [34].

According to the polarization plot of this sample in Figure 6, which does not show any passive layer, the R_ct_ depends on the formation of a corrosion product on the surface of the Inconel material, which is NiOH [35]. However, the Inconel material presented a higher corrosion resistance in different electrolyte solutions [1,36,37]. For the as-sprayed coating, the R_ct_ value was four times smaller than the value of the metallic substrate, meaning that there was a smaller charge transfer based on the ceramic layer chemical stability (Table 3); however, the R_s_ value was almost six times bigger. Considering the chemical inertia of ceramic materials in an electrolyte, in this case the YSZ coating, the R_ct_ value shown corresponds to the Inconel substrate because the electrolyte makes contact with the metal substrate through the interconnected pores of the ceramic coating.

For the Nyquist and Bode representations (Figure 7a,b), the fitting lines are very close to the real data points, with an error of less than 1%. 

The exponent n, which gives an indication of the deviation of the double electrical layer capacitance from ideality, is smaller in the Inconel + YSZ sample; this can be attributed to the increase in surface roughness due to the porosity of the layer. This behavior also suggests that although the reaction products formed during corrosion may be insoluble, they are likely to be porous and not a barrier to the reaction. In the Bode plot, the impedance at mid-frequencies represents the layer response, while at the lower frequency limit the process information is related to the substrate reaction at the electrolyte interface.

**Figure 8:**
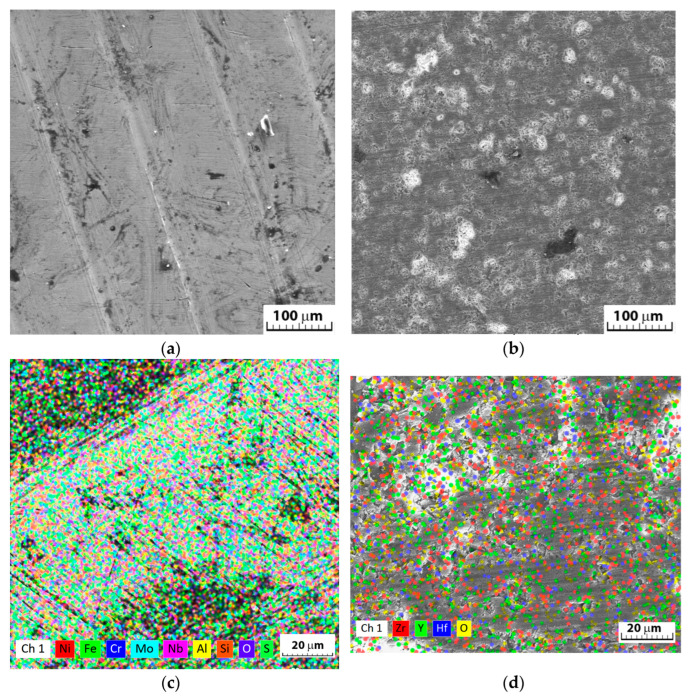
SEM images of the surface after the electro-chemical corrosion tests: (**a**) Inconel and (**b**) Inconel + YSZ and EDS mapping of the elements: (**c)** Inconel and (**d**) Inconel + YSZ.

The general aspect of the corrosion observed by cyclic potentiometry (Figure 5b) was confirmed by the SEM and EDS results, as no pitting was observed, nor were any sulphate-based compounds identified on the surface, although the element sulfur was identified on the surface of Inconel (Figure 8c) but in a very small amount (Table 3), probably as a deposit from the solution on the metal surface. The ceramic surface appeared to be unaffected in the H_2_SO_4_ solution (Figure 8b) with no pitting, surface compounds, or other deterioration. No percentage of S was detected on the surface of the ceramic coating, even though in some cases it was reported as material stuck in the pores from the surface [34]. The cyclic diagram potentiometry pattern was confirmed by the SEM images of the surface in Figure 8a.

As shown in Figure 8c, the compounds appearing on the surface, together with the oxidation state of the surface, contributed to the oxygen identification on the surface (Table 4) after corrosion at the same time as the decrease in Ni, Fe, and Cr percentages. As a result of the decrease in the percentages of these elements, an increase in the percentage of Nb on the surface was observed. Chemically, no influence of the electrolyte solution on the ceramic surface was observed, but a slight decrease in the oxygen percentage in favor of zirconium was observed when the values obtained in Table 1 and Table 4 were compared.

With an estimated market of more than USD 10 billion, the prospects for ceramic coatings to provide oxidation and erosion protection at high temperatures are endless. Industries that can benefit from the proper ceramic coatings include transport, energy, aerospace, maritime, and healthcare.

## 4. Conclusions

The main conclusions that can be drawn from the experimental results and the analysis performed are the following:

- New ceramic coatings of stabilized zirconia with 38 wt% Y_2_O_3_ powders were obtained by atmospheric plasma spraying on Inconel substrate (the ceramic coating showed good coverage of the surface substrate, using a NiAl-based bonding layer).
- The coated Inconel showed a lower corrosion potential in an acidic solution compared to the substrate sample (the ICORR value was 6.8 times lower for the coated sample). The corrosion resistance of the substrate was improved by the protective layer formed by the bonding material (deposited from Metco 410 powders) and the ceramics (grown from Metco 207 powders). As the ceramic coating was chemically inert, the role of this layer was to reduce the contact of the electrolyte solution with the anchoring layer (Al_2_O_3_ 30(Ni 20Al)), and the protection was achieved by the oxide layer at the interface between this anchoring layer and the ceramic layer.
- After the electrochemical corrosion tests, no cracks, pits, or compounds were observed on the coated sample, and the differences in the corrosion micro-morphologies of the surfaces (Inconel and Inconel + YSZ) were mostly caused by the galvanic coupling between the γ-matrix and the different secondary phases of the substrate.
- Based on the working environment (electrolyte solution), different bonding layers (chemical composition and thickness) can be proposed to improve the resistance of the ceramic coating.

## Figures and Tables

**Table 1:** Chemical compositions determined on the surface of the coating and also on several areas (point analysis mode on a spot area with 90 nm diameter) from Figure 3a.

Chem. Elem.	Zr %		Y %		O %		Hf %		Ni %		Fe %		Cr %		Al %		Nb %		Other %
	wt	at	wt	at	wt	at	wt	at	wt	at	wt	at	wt	at	wt	at	wt	at	
Coating Surface	44.9	20.7	29.2	13.8	25	65.4	0.9	0.2	-	-	-	-	-	-	-	-	-	-	-
Coating surface without bonding layer	43.1	19	29	13.1	26.9	67.7	0.9	0.2	-	-	-	-	-	-	-	-	-	-	-
Point 1 (substrate)	-	-	-	-	-	-	-	-	53.7	52.4	19.3	19.8	17.5	19.2	0.8	1.6	7.5	4.6	S῀1
Point 2 (bonding)	-	-	-	-	43.7	57	-	-	1.2	0.4	-	-	-	-	55	42.5	-	-	-
Point 3 (bonding)	16	6.9	10.1	4.5	21.7	53.2	-	-	51.6	34.5	-	-	-	-	0.6	0.9	-	-	-
Point 4 (coating)	48.8	24.4	29.1	14.9	21.2	60.4	0.9	0.2	-	-	-	-	-	-	-	-	-	-	-
EDS error %	1.7		1.2		5		0.2		1.5		0.6		0.5		1.5		0.2		

St dev. Zr: ±1.1; Y: ±0.5; O: ±2; Hf: ±0.1; Ni: ±0.5; Fe: ±0.2; Cr: ±0.2; Al: ±0.2; Nb: ±0.1; S: ±0.1.

**Table 2:** Electro-chemical corrosion process parameters.

Material	Corrosion Process Parameters					
	E (I = 0) (mV)	i_corr_ µA/cm	Rp (ohm.cm^2^)	v_corr_ (mm/year)	β_c_ (mV/dec)	β_a_ (mV/dec)
Inconel	−63.1	100.10	685.93	1.16	−452	435
Inconel + YSZ	−303.4 mV	14.61	3230	0.17	339	214

**Table 3:** Equivalent circuit parameters for Inconel and Inconel + YSZ in sulfuric acid solution.

	R_s_ Ohm.cm^2^	CPE		R_ct_ Ohm.cm^2^	CPE		R_pore_ Ohm.cm^2^
		Q Ss^n^/cm^2^	n		Q Ss^n^/cm^2^	n	
Inconel	68	3.89 × 10^−5^	0.78	4001	-	-	-
Inconel + YSZ	334	6.049 × 10^−5^	0.5	1007	24.64 × 10^−5^	0.7	1142

**Table 4:** Chemical composition of the surface of Inconel samples before and after the electro-chemical corrosion tests.

Elements/ Samples	Ni%		Fe%		Cr%		Nb%		Zr%		Y%		O%		Hf%		Others
	wt	at	wt	at	wt	at	wt	at	wt	at	wt	at	wt	at	wt	at	wt%
Initial Inconel	53.1	52.9	19.7	20.6	19.2	21.6	3.9	2.4	-	-	-	-	-	-	-	-	Mo: 4.2
Inconel after corrosion	49.2	35.5	18.2	13	17.4	14	4.1	1.9	-	-	-	-	1	1.3	-	-	Al: 0.5, S: 0.2
Inconel + YSZ coating	-	-	-	-	-	-	-	-	46.4	22.4	29.9	14.8	22.8	62.6	0.9	0.2	-
EDS error %	1.26		0.5		0.5		0.4		2.35		1.6		0.5–4		0.2		Mo: 0.01, Al: 0.07

St. dev.: Ni: ±1; Fe: ±0.75; Cr: ±0.2; Nb: ±0.1; Zr: ±1; Y: ±0.7, Hf: ±0.05; O: ±0.06; S: ±0.1, Al: ±0.01.

## Data Availability

The original contributions presented in this study are included in the article. Further inquiries can be directed to the corresponding authors.
